# Screening for Chemical Compliance in Water Quality Monitoring with *In Vitro* Bioassays

**DOI:** 10.1021/acs.estlett.6c00688

**Published:** 2026-09-08

**Authors:** Tessa E. Pronk, Miina Yanagihara, Corine J. Houtman, Renske P. J. Hoondert, Milo L. de Baat

**Affiliations:** † KWR Water Research Institute, Groningenhaven 7, 3433 PE Nieuwegein, The Netherlands; ‡ Het Waterlaboratorium, J.W. Lucasweg 2, 2031 BE Haarlem, The Netherlands; § Amsterdam Institute for Life and Environment A-LIFE, VU University, De Boelelaan 1108, 1081 HZ Amsterdam, The Netherlands; ∥ Department of Freshwater and Marine Ecology, Institute for Biodiversity and Ecosystem Dynamics, University of Amsterdam, Sciencepark 904, 1098 XH Amsterdam, The Netherlands

**Keywords:** water quality monitoring, bioassays, bioanalytical risk quotient percentage, compliance screening

## Abstract

Bioassays have proven their use in water quality monitoring. This study presents a novel framework for deploying bioassays to enable compliance screening in relation to regulatory threshold values. Bioanalytical equivalent concentrations for chemicals are derived from bioactivity data to calculate for each bioassay response the Bioanalytical Risk Quotient percentage (BRQ_%_): the percentage of chemicals potentially surpassing their quality standard (QS). If the bioassay response indicates BRQ_0_, none of the individual chemicals with known bioactivity in the bioassay surpass their QS. Should the bioassay response indicate potential QS exceedance by some candidates, the magnitude of that exceedance is a guide to prioritize chemicals for confirmation by instrumental analyses. Multiple bioassays are required to cover compliance for all relevant chemicals. As a proof of concept, compliance with the Dutch Drinking Water Regulation was evaluated for 100 surface water monitoring events by derivation of BRQ_%_ values for a battery of six bioassays. Full compliance could not be guaranteed for any of the water samples, with a median potential noncompliance for 13% of chemicals. The presented framework has potential for broader application, such as compliance testing related to QS for human health or the environment in a variety of matrices or for specific permits.

## Graphical abstract

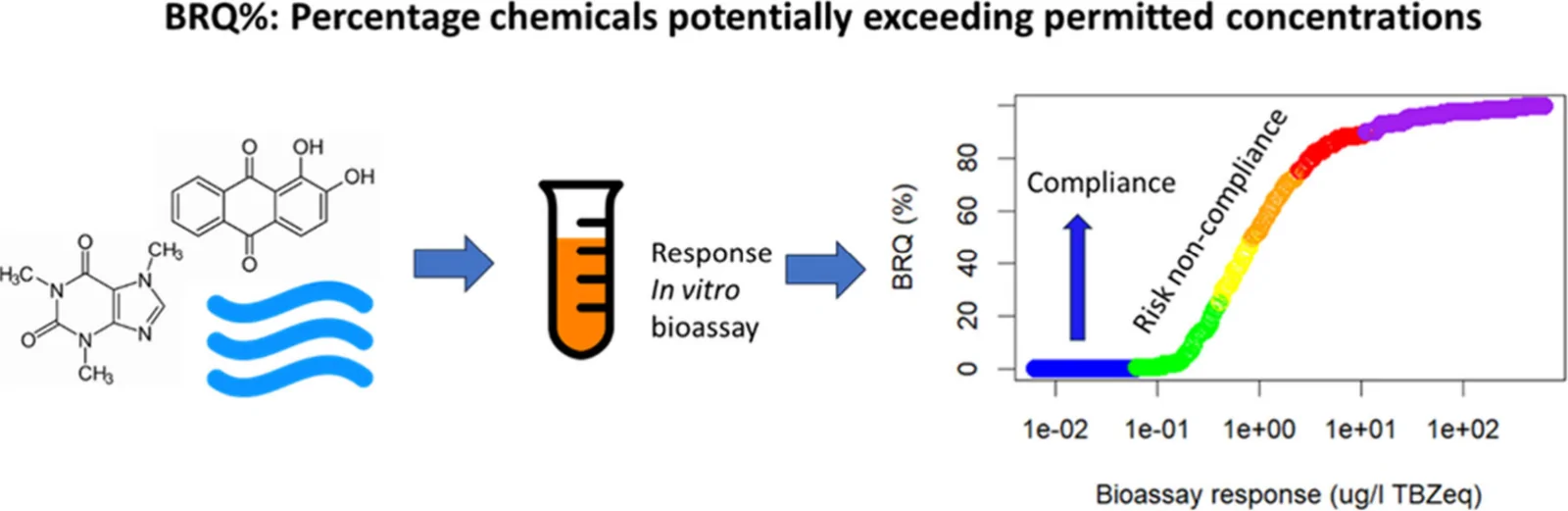

## Introduction

Clean drinking water is essential for human health.^1,2^ The European Drinking Water Directive (EU DWD)^3^ sets strict chemical quality standard (QS) values for drinking water sources. This allows compliance testing for many chemicals, including contaminants of emerging concern,^2^ even before drinking water is produced, while simultaneously making this evaluation a substantial task.

In general, chemical compliance monitoring for matrices of unknown chemical composition is carried out using targeted analytical chemical methods, which detect individual chemical concentrations.^4^ Because of the potentially wide variety of relevant chemicals, analysing all is costly and time-consuming. *In vitro* bioassays have demonstrated their ability to sensitively detect the specific effects induced by chemicals (e.g., refs 5 and 6 ). In combination with sample concentration, this may lead to detectable bioassay responses (far) below exposure concentrations that are relevant for human health effects.^7^ Moreover, bioassays can detect an infinite range of chemicals, if they trigger the mode of action of the bioassay.

Bioassays support safe drinking water production in several specific ways.^6,8,9^ There is broad support for their application in assessing the suitability of new water sources, indicating possible risks, evaluating treatment efficacy, or for routine water monitoring to identify changes in measured effects. The use of bioassays as a water quality measure with a set numerical target (i.e., guideline value or QS), however, was, thus far, out of scope. This study proposes an alternative perspective on the use of bioassays. We argue that bioassays can serve as tools for explicit testing of compliance with chemical QS. This new paradigm extends the use of bioassays from effect-based water quality monitoring to compliance testing.

In the proposed approach, bioactivity data from individual chemicals are used to define the potential maximum concentrations of each of these chemicals corresponding to an observed bioassay response. Hence, the analysis works from a ‘worst case’ scenario in which a single chemical induces the bioassay response. This is used to calculate a Bioanalytical Risk Quotient percentage (BRQ_%_), which is defined as the percentage of chemicals potentially surpassing a QS. BRQ_%_ values are thus explicit risk estimates over the entire range of possible bioassay responses, rather than distinguishing between a response above or below a single trigger value (e.g., refs 10–12 ). The approach also identifies *which* chemicals may exceed QS and to what extent, allowing for the prioritization of chemicals for confirmation by instrumental analyses.

Although currently this approach is presented as a proof-of-concept and needs further testing in practice, it could make compliance testing more efficient. Analytical verification is required only for the chemicals potentially exceeding their QS, strongly narrowing down the number of targets for subsequent chemical analytical compliance testing. If the bioassay response indicates a BRQ_%_ of 0, no chemicals with bioactivity in the bioassay exceed their corresponding QS values. No further chemical analytical verification is required. Hence, BRQ_0_ would be a logical numerical regulatory target based on effect-based measurements. Using a battery of bioassays representing complementary end points resulting in a wide chemical coverage is essential in effect-based water quality assessment,^13^ and vital to the framework. Bioassay-specific BRQ_%_ values can be combined into a BRQ_%_ per sample, integrating the outcomes of all bioassays in the battery into a single value.

To demonstrate the proposed framework, it is applied to assess (risk of non) compliance with the Dutch Drinking Water Regulation (Dutch DWR)^14^ QS in sources for drinking water production.

## Materials and Methods

### Chemical QS for Assessing Compliance with the Dutch DWR

In addition to (health-based) QSs for specific chemicals and ‘pesticides’ (0.1 μg/L), the Dutch DWR prescribes QSs for chemicals labelled ‘anthropogenic’ (1 μg/L) as a signal to follow up on their health risks. The registry of water utility water-quality data (REWAB) contains labels to assign the correct QS values to chemicals.^15^ Via their REWAB labels, QS values were linked to 2228 chemicals with a CAS registry number.

### Bioactivity Data Collection and Bioassay Selection

Bioactivity data (‘concentration-response curve’) for individual chemicals in bioassays are essential to calculate BRQ_%_ values. An extensive collection is available via the ToxCast database.^4^ Combining assays into a battery covering a broad range of toxic mechanisms and chemicals is common practice in effect-based water quality assessment.^13,16^ Here, we selected six bioassays that, firstly, represent effect types proposed for inclusion in bioassay batteries,^13,17^ and, secondly, for which the ToxCast database contained ample bioactivity data (see SI III) for chemicals with a QS in the Dutch DWR (Table 1).

**Table 1:** Selected Bioassays from the ToxCast Database^4^

Assay name	Short assay name	Specific end point tested	Effect type	Unique bioactive chemicals	Dutch DWR chemicals
ATG_NRF2_ARE_CIS_up	Nrf2	Nuclear factor erythroid 2-related factor 2 upregulation	Induction of adaptive stress response	727	190
TOX21_ERa_LUC_ VM7_Agonist	ERα	Estrogen receptor alpha activation	Endocrine disruption	758	127
TOX21_RT_HEK293_GLO_40 h_viability	Cytotoxicity	Cell viability	Apical effect	825	183
TOX21_PXR_Agonist	PXR	Pregnane X Receptor activation	Xenobiotic metabolism	1139	248
TOX21_DT40_100	DNA damage	DNA repair	DNA damage	1410	261
TOX21_PR_BLA_ Antagonist_ratio	Anti-PR	Progesterone receptor antagonism	Endocrine disruption	907	208

In total, 493 of 2228 chemicals with a QS in the Dutch DWR have demonstrated bioactivity in one or more of the six selected ToxCast bioassays.

### *In Silico* Bioassay Responses in Dutch Surface Waters

Compliance calculations start with bioassay responses measured in a monitoring event. However, such data are not available for the selected assays. Therefore, SI I explains the calculation of *in silico* bioassay responses used to demonstrate the framework and illustrate the interpretation of compliance testing with bioassays in this study.

### Calculating Bioassay-Based Compliance

A stepwise calculation framework was applied to translate bioassay responses into indicators of regulatory compliance. The starting point for calculations is a measured bioassay (‘*j*’) response, expressed in a reference compound (‘*y*’) bioequivalent concentration; *BEQ*_*yj*_. All bioassay responses were expressed in thiabendazole equivalents (μg/L TBZeq), because it was bioactive in all selected bioassays. This facilitates a straightforward comparison of the BRQ_%_ vs bioassay response relations across bioassays. In practice, however, different reference compounds can be used.

The bioassay response expressed in *BEQ*_*yj*_ can be recalculated to equivalent concentrations of other individually tested chemicals *i* in bioassay *j*, under the assumption that *i* alone induces the response, with the help of relative effect potencies (REP).

1 $${BEQ}_{ij}=\frac{{BEQ}_{yj}}{{REP}_{ij}}$$

Here, *BEQ*_*ij*_ is for chemical *i*, bioassay *j*. *REP*_*ij*_ is based on available bioactivity data (eq 2).

2 $${REP}_{ij}=\frac{{AC50}_{yj}}{{AC50}_{ij}}$$

Here, *AC50*_*ij*_ is the concentration at which half of the maximum activity in bioassay *j* is reached for chemical *i*, and *AC50*_*yj*_ represents this value for the reference chemical *y* in bioassay *j*. A high REP implies that a relatively low concentration can induce the bioassay response.

A ‘risk quotient’ (RQ) indicates how the *BEQ*_*ij*_ of a chemical relates to its corresponding QS.

3 $${RQ}_{ij}=\frac{{BEQ}_{ij}}{{QS}_{i}}$$

In this equation, *RQ*_*ij*_ is the risk quotient of chemical *i* at *BEQ*_*ij*,_ concentration, and *QS*_*i*_ is the quality standard value for chemical *i*. An RQ >1 implies that, at the measured bioassay response, the *BEQ*_*ij*_ exceeds QS.

Using eqs 1, 2, and 3, RQ values of all chemicals with bioactivity data and QS can be calculated for any bioassay response. The percentage of chemicals exceeding the RQ of 1 (BRQ_%_) – is used as an indicator of the risk of noncompliance of bioactive chemicals in bioassay *j* exceeding their QS at a given bioassay *j* response in BEQ_yj_ (eq 4).

4 $$B{RQ}_{j\%}=\frac{\mathrm{number of chemicals bioactive in}\;j\;\mathrm{with}\;RQ>1\;\mathrm{in}\;j\;\mathrm{at}\;{BEQ}_{yj}}{\mathrm{number of chemicals bioactive in}\;j}•100$$

To facilitate interpretation, colours are used to classify different ranges of BRQ_%_ values. Blue: full compliance (BRQ_0_; i.e., no chemical concentration exceeds its QS); green: up to 25% potential noncompliance; yellow: 25–50%; orange: 50–75%; red: 75–90%; and purple: >90% (Figure 1).

**Figure 1:**
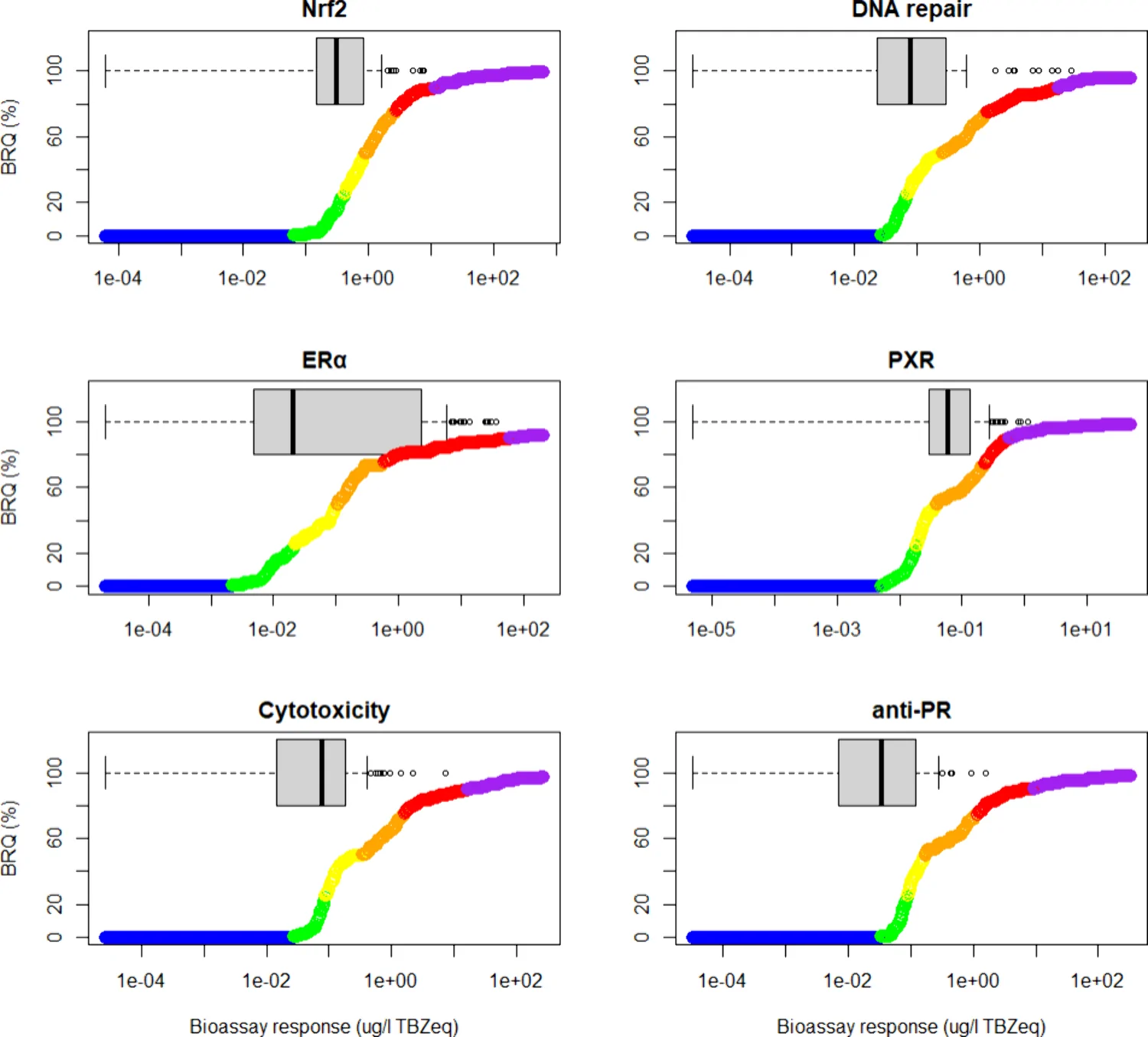
BRQ_%_ - bioassay (Table 1) response relationships. Boxplots depict (*in silico*) bioassay responses (SI I) in Dutch surface water samples. Colours indicate the BRQ_%_ compliance class (see Materials and Methods).

If individual chemical BEQ concentrations required to induce the measured bioassay response exceed their QS this can be seen as a (worst case) risk, and certainly not all will exceed. In practice, the bioassay response may also be a result of a mixture of chemicals where each chemical concentration stays below its corresponding QS. The bioassay response may also be induced by a chemical that does not fall under the applicable regulation, but such chemicals are out of scope for compliance testing.

### Integrating Bioassay Battery BRQ_%_s

Bioassays are complementary in their sensitivity to chemicals. Some chemicals will have seemingly high potential concentrations because of a lack of sensitivity of the bioassay. Compliance of chemicals per bioassay is therefore best assessed considering all bioassays. If RQ values differ between bioassays applied to the same sample, the most sensitive bioassay logically determines the maximum possible chemical concentration, and thus the lowest RQ. This means that the BRQ_%_ value for a bioassay may prove lower if other bioassay RQs are considered. The adjusted BRQ_%_ value (BRQ_jadj%_) for a bioassay *j* in a battery with *n* other bioassays, applied to the same water sample, is calculated as

5 $$B{RQ}_{jadj\%}=\frac{\mathrm{number of chemicals bioactive in}\;j\;\mathrm{with}\;RQ>1\;\mathrm{in}\;j-n\;\mathrm{at}\;{BEQ}_{yj-n}}{\mathrm{number of chemicals bioactive in}\;j}•100$$

$B{RQ}_{jadj\%}$ is the percentage of chemicals bioactive in bioassay *j* that remain potentially noncompliant, i.e., have RQ > 1 over all *n* bioassays.

An overall decision on chemical compliance requires an integrated overall BRQ_%_ score for a monitoring event, considering *all* chemicals with bioactivity data and QS in a bioassay battery. For this purpose, the lowest RQ for each chemical bioactive in any bioassay is used to calculate the combined BRQ_%_ value (BRQ_comb%_) based on:

6 $$B{RQ}_{comb\%}=\frac{\mathrm{number of chemicals bioactive in}\;j-n\;\mathrm{with}\;RQ>1\;\mathrm{in}\;j-n\;\mathrm{at}\;{BEQ}_{yj-n}}{\mathrm{number of chemicals bioactive in}\;j-n}•100$$

$B{RQ}_{comb\%}$ is the percentage of all chemicals bioactive in any bioassay of the battery that have an RQ >1 over all *n* bioassays in the same sample. It can be referred to as the overall BRQ_%_ for a sample.

An example for the calculation of BRQ_jadj%_ and BRQ_comb%_ is provided in SI II. A set of potential questions on the BRQ framework is addressed in SI IV.

## Results

Equations 1–4 link bioassay responses to potential concentrations of chemicals in a sample. BRQ_%_ values were plotted in relation to bioassay responses in μg/L TBZeq (Figure 1). This relation is fixed and can be used to look up BRQ_%_ values for measured bioassay responses. The ranges of (*in silico*) bioassay responses (μg/L TBZeq) in Dutch surface waters (Supplement I) are indicated as boxplots (Figure 1).

The BRQ_%_ for the individual water samples ranged from the blue to the purple classes. The medians of the *in silico* bioassay responses for samples (see boxplots, Figure 1) correspond to BRQ_%_ in the green (for Nrf2, ERα, anti-PR, Cytotoxicity), yellow (DNA damage), or orange class (PXR) (Figure[Fig fig1]).

BRQ_jadj%_ values for the surface water samples were calculated using eq 5 (Figure 2). Finally, the bioassay battery results were combined into single BRQ_comb%_ values (eq 6) (rightmost boxplot, Figure 2), integrating potential noncompliance for all chemicals that are bioactive in the battery. The reduction in BRQ_%_ values from BRQ_j%_ to BRQ_jadj%_ and to BRQ_comb%_ is obvious (Figure 2).

**Figure 2:**
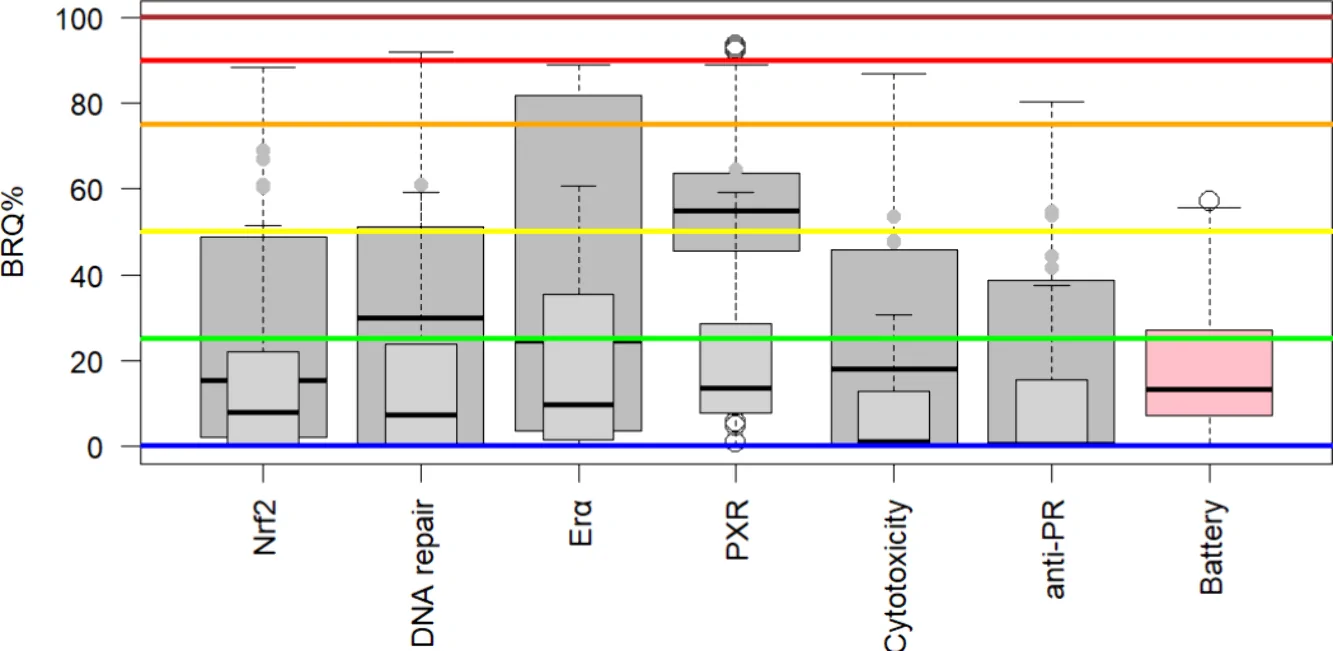
Distribution of BRQ_j%_ values for water samples in gray (broad) boxplots, with outliers shown as empty circles. The adjusted BRQ_jadj%_ distribution is overlayed in light-gray (narrow) boxplots, with outliers shown as filled gray points. The right-most boxplot is the distribution of the BRQ_comb%_ values, considering all chemicals in all selected bioassays (Table 1). Horizontal colored lines represent the risk classes (see Materials and Methods).

None of the *in silico* effect-based monitoring events are in the fully compliant blue class (BRQ_comb%_ = 0), 73 are in the green class (BRQ_comb%_<25), 21 are in the yellow class (BRQ_comb%_<50), and 6 are in the orange class (BRQ_comb%_<75) (not shown). The median BRQ_comb%_ implies that 13% of the available chemicals included in this study potentially exceed their corresponding DWR QS, given the current applied bioassay battery and median *in silico* bioassay responses. Since compliance is required for 2228 chemicals, 290 chemicals (13%) would require follow-up through chemical analytical measurements, in a scenario where bioactivity data are available for all 2228 chemicals.

## Discussion

The proof of concept demonstrates that the bioassays included in this study together indicate a moderate risk of noncompliance with the Dutch DWR for surface water samples, with a median BRQ of 13% of chemicals potentially surpassing their QS. The BRQ percentage may prove to be lower if, in the future, complementary bioassays are identified that respond more sensitively to chemicals than the bioassays currently included.

Extensive monitoring programs with chemical target analyses in the Netherlands show that multiple chemicals indeed regularly exceed QS in practice.^1^ The proposed framework provides a quantitative link between bioassay responses and regulatory limits, allowing the identification of potential exceedances. The calculated maximum magnitude of RQs supports prioritisation of chemicals for confirmation analysis and complements existing chemical analytical and effect-based monitoring programmes. Any follow-up measures when results indicate noncompliance can be targeted and economical, with a limited number of candidate analytes. When bioassays are applied for chemical water quality monitoring, this framework allows the same data to be used for compliance testing, adding substantial value to the application of bioanalytical tools. The proposed approach is not limited to assessing compliance with QS related to drinking water sources; it also encompasses compliance with thresholds in other matrices like drinking water itself, surface water, food, or air, defined functional groups of chemicals, or chemicals listed in discharge permits. The resulting compliance will differ with the strictness of the chosen QS.

Bioassays are repeatedly mentioned as promising in different regulatory contexts, like the World Health Organization (WHO) guidelines for potable reuse^18^ and, more recently, the European Commission agreement to update the European Water Framework Directive, which now requires the use of effect-based methods to monitor effects of harmful chemical mixtures in surface waters, particularly for estrogenic chemicals.^19^ Since 2018, the California State Water Board has required the use of ERα and AhR bioassays for monitoring the quality of recycled water intended for indirect potable reuse.^20^ The proposed framework enables compliance testing using bioassays, with a numerical target threshold, BRQ_0_. This may facilitate further implementation of bioassays as bioanalytical tools, as well as the legal embedding of effect-based water quality monitoring strategies.^6,7^

## Supplementary Material



## Notes

Explanation of the calculation of *in silico* bioassay responses (SI I). Illustration of the calculation of adjusted and combined BRQ% (SI II). ToxCast hit call rules (SI III). Question and answers on the BRQ_%_ framework (SI IV). (PDF)

## ⊥

National Institute for Public Health and the Environment (RIVM), Antonie van Leeuwenhoeklaan 9, 3721 MA Bilthoven, The Netherlands
