# Supplementary material for: Screening for Chemical Compliance in Water Quality Monitoring with In Vitro Bioassays
Source: Environ Sci Technol Lett. 2026 Sep 8;13(10):1314–9. doi: 10.1021/acs.estlett.6c00688 (PMC13671473; doi:10.1021/acs.estlett.6c00688)
Supplement: Supplementary file 1 [file ez6c00688_si_001.pdf]

Supporting Information (SI) I-IV: Screening for Chemical Compliance in Water Quality Monitoring with In Vitro Bioassays

Tessa E. Pronk[1], Miina Yanagihara[1], Corine J. Houtman[2,3], Renske P.J. Hoondert[1], Milo L. de Baat[4]*

1. KWR Water Research Institute, Groningenhaven 7, 3433 PE Nieuwegein, the Netherlands

2. Het Waterlaboratorium, J.W. Lucasweg 2, 2031 BE Haarlem, the Netherlands

3. Amsterdam Institute for Life and Environment A-LIFE, VU University, De Boelelaan 1108, 1081 HZ Amsterdam, the Netherlands

4. Department of Freshwater and Marine Ecology, Institute for Biodiversity and Ecosystem Dynamics, University of Amsterdam, Sciencepark 904, 1098 XH Amsterdam, the Netherlands

* Corresponding author

Contents

SI I Calculating in silico bioassay responses 2

SI II Illustration of the calculation of adjusted and combined BRQ% 4

SI III ToxCast hit call 8

SI IV BRQ% framework Question & Answer 8

SI I Calculating in silico bioassay responses

To evaluate the applicability of the developed framework in real-life chemical water quality assessment scenarios, *in silico* bioassay responses in Dutch surface waters were calculated based on measured chemicals' concentrations from available chemical analytical water quality monitoring data. Although it would have been better to use actual measured bioassay responses in water samples for the bioassays used in the current study, this data is not available, because Dutch surface waters are not monitored with these specific bioassays, but many of them with analogue assays for the same endpoints (Houtman et al., 2025). For these analogous bioassays, chemicals' bioactivity data are generally not publicly available.

The *in silico* bioassay response for bioassay j expressed in reference compound y concentration (expressed as BEQ_{yj}, for the proof of concept in this manuscript in µg/L thiabendazole) was calculated based on available monitoring concentration data using Equation S1 (Escher, Neale and Leusch, 2021):

$$BEQ_{yj} = \sum C_i \cdot REP_{ij} \qquad \text{Equation S1}$$

C_i is the analytically measured chemical concentration for chemical i. REP_{ij} is the relative effect potency of the chemical compared to the reference compound y, which was derived for each chemical i in bioassay j based on available bioactivity data. Equation S1 assumes that all chemicals follow the concentration addition (CA) model when considering mixture effects.

Chemical concentration data were obtained from the Dutch 'Waterkwaliteitsportaal' (WKP, 2024) for the hundred monitoring events in surface waters with the highest number of measured parameters in 2023, which was the most recent available year. These waters are managed by different water authorities across the Netherlands: Rijn & IJssel, Zuiderzeeland, Vallei en Veluwe, Vechtstromen, and Drents Overijsselse Delta. Per monitoring event, chemical concentrations for 474 to 556 unique chemicals were reported. Bioactivity data for the bioassays in this study was available for a percentage of these chemicals per event, mean and standard deviation depending on the bioassay, Nrf2: 17% (+- 0.6), DNA damage: 17% (+-0.2), ERα: 8% (+-0.2), PXR: 16% (+-0.6), Cytotoxicity: 11% (+-0.2), anti-PR: 13% (+-0.4). However, part of these chemicals may be missing because they do not exert a response in the bioassay (they are 'inactive'), and adding such chemicals would not increase the calculated bioassay response.

In many cases, chemicals were below their minimum reporting limit. In these cases, the concentrations were set to zero. For this reason, and for the fact that bioactivity data were not available for all monitored chemicals, the *in silico* bioassay response most probably is an

underestimation of the actual bioassay response that these water samples would evoke. Because we provide a proof-of-concept, the *in silico* bioassay responses are used as a best guess with regards to bioassay responses and, also important, their ratio, for water samples containing relevant chemicals, to illustrate the results and interpretation of the BRQ% framework.

Escher, B., Neale, P., Leusch, F. (2021) Bioanalytical Tools in Water Quality Assessment. https://doi.org/10.2166/9781789061987 ISBN (electronic): 9781789061987 IWA Publishing.

Houtman, C.J., van der Neut-Marchand, C., van Oorschot, Y.G.M., Lamoree, M.H., Steen, R.J.C.A. (2025) Combination of effect-based monitoring and targeted screening of (drinking)water II: Effect directed analysis of compounds responsible for bioassay activity. Environ Int. 205:109782. doi: 10.1016/j.envint.2025.109782.

SI II Illustration of the calculation of adjusted and combined BRQ%

As an illustration of the calculation of the $BRQ_{jadj\%}$ an example calculation is given in this supplement. The example calculation is done for the ERα bioassay. For the required bioassay responses, a fictive water monitoring event is assumed. To obtain bioassay responses of a realistic magnitude, the median of calculated *in silico* bioassay responses (see SI I) are taken. In Figure 1 in the manuscript the median response is indicated as a thick vertical line in the boxplots per bioassay. RQ for individual chemicals, based on these bioassay responses, are calculated with Eq. 1-3 in the manuscript.

For example, the calculation of the RQ of imazaquin for the ERα bioassay (Table A) with Eq. 1-3 is as follows. The fictive monitoring events' ERα bioassay response is 0.0211 µg/L thiabendazole (reference chemical, TBZ) equivalents (shown as 0.02 in Table A). The REP of imazaquin is calculated from the ERα bioassay AC_{50} values provided in ToxCast. For thiabendazole this is 0.987 mg/L and this is divided by the ERα bioassay AC_{50} value of imazaquin (29.88 mg/L). This results in 0.987 / 29.88 = 0.033 (Eq.2). A low REP means that a chemical is less potent than the reference compound; a higher concentration is required to induce the given response in the ERα bioassay. Therefore, the potential maximum concentration of imazaquin, under the assumption that imazaquin induces the bioassay response as a single chemical (a worst-case scenario) is the thiabendazole concentration divided by the REP of imazaquin; 0.0211 / 0.033 (Eq.1) and this amounts to 0.64 µg/L. The QS in the Dutch DWR is 0.1 µg/L and therefore the RQ is 0.64 / 0.1 = 6.4 (Eq.3); the concentration of imazequin potentially exceeds the QS by 6.4 times. This calculation is done for all RQ shown in Table A.

In total, 31 chemicals have RQ>1 based on the example ERα bioassay response. On a total of 127 chemicals with bioactivity data in the ERα bioassay (see Table 1 in the manuscript) this yields a $BRQ_{\%}$ of 24.4% (e.g. 31 out of 127). In the current proof of concept, the other selected bioassays (Table A) have RQ for some of these chemicals, the height of which is based on the example bioassay response. For 18 of 31 chemicals of the ERα the minimum RQ in these other selected bioassays is ≤1 (lower part of Table A, column min RQ, indicated in grey). The RQ for a chemical can be lower in any bioassay in the used bioassay battery if the chemical is more potent to evoke a response, making that bioassay more suitable to

detect lower concentrations of the chemical. Several chemicals in the ERα do not exceed their QS according to the other bioassays in Table A and the chemicals can consequently be disregarded in a recalculation of the $BRQ_{\%}$ value for the ERα bioassay. Considering this, Table A shows that now 13 out of 127 chemicals bioactive in the ERα are still potentially exceeding their QS. This yields a $BRQ_{jadj\%}$ of 10.2% for the ERα bioassay. This reduction of the BRQ from 24.4% to 10.2% can also be observed in Figure 2 in the manuscript, by comparing the horizontal lines of the large and small boxplot for ERα.

Table A. Illustrative example. Chemicals with RQ>1 in the ERα bioassay for bioassay responses in a fictive example surface water monitoring event. The bioassay responses correspond to the median of responses in Dutch surface water samples (see boxplots of Figure 1 in the manuscript). Empty cells imply that the RQ could not be calculated because the chemical is not bioactive or AC50 data was not available in the ToxCast database. Grey rows indicate chemicals with RQ>1 in the ERα and RQ<1 in any of the other bioassays.

		Nrf2	DNA damage	ERα	PXR	Cytotoxicity	Anti-PR	Minimum RQ
Example bioassay responses		0.31 µg/L TBZeq	0.08 µg/L TBZeq	0.02 µg/L TBZeq	0.06 µg/L TBZeq	0.08 µg/L TBZeq	0.03 µg/L TBZeq	
CAS number	Chemical name	RQ						
81335-37-7	Imazaquin			6.40				6.40
626-43-7	3,5-Dichloroaniline			2.99				2.99
107-49-3	Tetraethyl pyrophosphate			2.77				2.77
579-66-8	2,6-Diethylaniline			2.42				2.42
41394-05-2	Metamitron			2.15				2.15
3060-89-7	Metobromuron		1.90	1.99	5.16			1.90
122-42-9	Propham			1.67	7.76			1.67
35256-85-0	Butam		1.66	2.50				1.66
34123-59-6	Isoproturon		1.63	2.50	2.63			1.63
34014-18-1	Tebuthiuron	1.44		9.88	3.74			1.44
123312-89-0	Pymetrozine			1.30				1.30
18854-01-8	Isoxathion			1.17				1.17
1563-66-2	Carbofuran		1.10	3.73	4.70			1.10
95-57-8	2-Chlorophenol	0.98		1.04				0.98

300-76-5	Naled		0.96	4.83	7.04	2.51		0.96
150-68-5	Monuron		0.95	2.60				0.95
23135-22-0	Oxamyl	0.72	1.25	2.76				0.72
103-33-3	Azobenzene		0.70	1.86				0.70
1596-84-5	Daminozide	0.64		1.03				0.64
13684-56-5	Desmedipham	0.55		1.43		0.92		0.55
27314-13-2	Norflurazon	0.50	2.11	6.55	6.13	2.22	0.68	0.50
74115-24-5	Clofentezine	0.44		1.03	1.09			0.44
298-02-2	Phorate		1.03	3.11			0.36	0.36
43121-43-3	Triadimefon	0.76	0.99	1.24	9.15	0.93	0.35	0.35
298-00-0	Methyl parathion		1.56	3.27			0.30	0.30
126833-17-8	Fenhexamid	0.29		2.08	2.20		0.38	0.29
59-50-7	4-Chloro-3-methylphenol			1.37			0.21	0.21
17804-35-2	Benomyl		0.21	3.10	4.40	0.37	0.52	0.21
52-68-6	Trichlorfon	0.92		2.37	3.59		0.14	0.14
10605-21-7	Carbendazim	0.46	0.13	2.02		0.09		0.09
133-06-2	Captan		0.02	1.14	0.56	0.74	0.37	0.02

The calculation of the overall $BRQ_{comb\%}$ is conducted based on RQ values derived for all example bioassay responses and chemicals with bioactivity data. The calculation is quite simple. The minimum RQ of a chemical over all included bioassays determines if a chemical is potentially above or below an RQ of 1.

An example with all chemicals would result in a very large table, therefore a toy example will illustrate this more efficiently. For the following toy example of 25 chemicals in Table B the minimum RQ (last column) is determined across all bioassays for each chemical in this random selection of 25 chemicals. As shown in Table B, the minimum RQ exceeds ‘1’ for five out of the 25 chemicals. This corresponds to $BRQ_{comb\%}$ of 20% for this water sample, as evaluated by this bioassay battery. The individual $BRQ_{\%}$ for the bioassays (count all RQ>1, divide by number of chemicals with bioactivity) in this toy example are, respectively: Nrf2 39% (7/18), DNA damage 53% (9/17), ERα 29% (2/7), PXR 94% (15/16), Cytotox 46% (6/13), anti-PR 0% (0/14).

Table B. Illustrative example. RQ for a subset of 25 chemicals for the median (in silico) bioassay responses in Dutch surface water samples. Empty cells mean the RQ could not be

calculated because the chemical is not bioactive or AC_{50} data was not available in the ToxCast database. Grey rows indicate chemicals with RQ<1 in any of the bioassays.

		Nrf2	DNA damage	ERα	PXR	Cyto-tox	Anti-PR	Min RQ
**Example bioassay responses**		**0.31 µg/L TBZeq**	**0.08 µg/L TBZeq**	**0.02 µg/L TBZeq**	**0.06 µg/L TBZeq**	**0.08 µg/L TBZeq**	**0.03 µg/L TBZeq**	
**CAS number**	**Chemical name**	**RQ**						
18854-01-8	Isoxathion			1.17				1.17
32809-16-8	Procymidone	0.15			2.91		0.37	0.15
1982-49-6	Siduron				2.29	0.75	0.35	0.35
18691-97-9	Methabenzthiazuron	0.46	0.85	0.15	3.72		0.44	0.15
86-29-3	Diphenylacetonitrile	1.77				0.46		0.46
110235-47-7	Mepanipyrim	1.83	0.93	0.98	2.56	0.13	0.25	0.13
950-37-8	Methidathion	0.93	1.88				0.51	0.51
79-57-2	Oxytetracycline	4.97	2.28		2.94			2.28
57837-19-1	Metalaxyl	1.70	2.29		10.16			1.70
13684-63-4	Phenmedipham			0.42	1.77			0.42
60168-88-9	Fenarimol	0.26	0.59	0.97	3.92	0.60	0.20	0.20
934-32-7	2-Aminobenzimidazole		1.08					1.08
110488-70-5	Dimethomorph	0.43	1.60		1.61	1.16	0.60	0.43
142459-58-3	Flufenacet	0.78	0.60		1.84			0.60
63-25-2	Carbaryl	0.47	1.01		3.49	0.11	0.27	0.11
55814-41-0	Mepronil		1.20		1.67	1.06	0.34	0.34
7287-19-6	Prometryn	0.27	1.68		1.81	1.09	0.46	0.27
51-28-5	2,4-Dinitrophenol	0.90	1.16					0.90
13194-48-4	Ethoprop	1.03						1.03
24017-47-8	Triazophos					1.59	0.44	0.44
95-57-8	2-Chlorophenol	0.98		1.04				0.98
1982-47-4	Chloroxuron		0.42		1.64	0.94		0.42
68694-11-1	Triflumizole	0.25	0.55		2.60	1.44	0.13	0.13
534-52-1	2-Methyl-4,6-dinitrophenol	1.74	0.81			1.21	0.18	0.18
148-79-8	Thiabendazole	3.11	0.80	0.21	0.62	0.77	0.34	0.21

Some additional remarks are relevant. Firstly, in this example, the PXR bioassay response is quite high, resulting in a high BRQ% for this bioassay (15 out of 16 chemicals have RQ>1, which would be a BRQ_{94} and would fall in the very high risk 'purple' class. When taking into consideration the other bioassay responses, 14 out of 16 PXR chemicals in Table B are

actually proving compliant (minimum RQ<1), and this results in a BRQ_{adj13} for this bioassay. This shows the vital advantages of using a bioassay battery. The interpretation, in other words, is that the PXR bioassay response can be induced at concentrations that are above RQ by 15 of the 16 chemicals, but given the bioactivity and response in other bioassays, QS is in practice potentially exceeded by only two of these chemicals.

Secondly, the maximal potential RQ in this toy example is for the chemical oxytetracycline and is 2.28 times the QS concentration value. Having specific RQ available thus allows for a critical reflection of the potential exceedance magnitude, and this can be taken into account in choosing whether risks are high enough to merit follow-up analytical chemical analyses.

SI III ToxCast hit call

For the AC_{50} data of the ToxCast database, the hit-call for a chemical in a bioassay was considered active when:

1. The Hill model should emerge as the model with the best fit for the dose-response (based on lowest AIC – Akaike Information Coefficient)

2. The top of the modeled curve was above the efficiency threshold (efficacy cutoff, the maximal experimental value on which the model was based)

3. For at least one concentration, the AC_{50} (median response) was above the efficacy threshold

SI IV BRQ% framework Question & Answer

The novel BRQ% framework in this paper is explained in quite a concise way. The following Q&A were formulated based on the helpful comments of three reviewers. These questions help to better understand the framework.

Interpretation

If chemicals have RQ>1 in the framework, are these indeed exceeding their quality standard (QS) concentration?

The calculated RQ is potential, considering in a worst case when a chemical alone induces the measured bioassay response. Thus, chemical analytical analyses will have to rule out an actual exceedance of QS. The other way around, the framework is set up in such a way that if chemicals have a calculated RQ<1, the chemical indeed is below its QS. Therefore, if all chemicals of interest have a calculated RQ<0 (BRQ_0), none exceed their QS, in principle.

Is a water sample with BRQ_0 ground to discard further chemical analyses required by regulation?

No, until the BRQ framework enters regulation, regulatory-required analyses remain mandatory. If the monitoring event is not done under a regulatory requirement, however, the BRQ_0 could, in theory, be sufficient for water managers to assess the chemicals that are evaluated. To enable this, the BRQ framework first needs practical validation to go from a proof of concept as described in this paper, to a working methodology.

Which chemicals are not covered in the framework?

Chemicals that are relatively non-toxic and show no activity in any bioassay will still need verification with chemical analyses because these are not included in the calculation of BRQ_0, as no potential maximum concentration can be calculated. If a limited set of bioassays is included in practice, the same goes for chemicals that *are* toxic but do not show bioactivity in the selected bioassays.

If for the same monitoring event one bioassay indicates concentrations of a chemical higher than QS, and another bioassay lower than QS, what can be concluded?

The most sensitive bioassay is leading; the potential concentration of a chemical can only be as high as indicated by the most sensitive assay.

Quality standard

What quality standard (QS) should I use in the framework?

Any relevant QS for which compliance testing is required can be used. In the article, we chose to showcase compliance with the Dutch drinking water regulation, which is a mixture of standards for surface water intended for drinking water production, based on health, organoleptic quality, and signaling for further inquiry, falling under a single regulation. In practice, even multiple QS can be evaluated (e.g. for surface water, both ecological and humane-health based QS could be relevant) and chemical compliance can be proven for one, both, or none.

Can the framework detect potential concentrations of chemicals with low regulatory QS?

Yes, it can. The QS does not influence the ability of the framework to calculate maximum potential chemical concentrations. However, the detection limits depend on the sensitivity of the applied bioassay.

Can the framework detect concentrations below QS for all chemicals, in practice?
Not by default. This depends on the (relative) sensitivity of the selected bioassays for the chemical and the strictness of the QS. Low QS are exceeded more easily. If QS are very low, samples may more readily fall in the 'red' or 'purple' class. Moreover, chemicals that show no activity in any of the selected bioassays cannot be evaluated.

Does the choice of quality standard (QS) set impact the choice of bioassays?
If the QS are relatively low, bioassays will logically indicate a higher potential for non-compliance. If the most sensitive bioassays for the chemicals are already integrated in the bioassay battery, there is no advantage to change the bioassay selection. If the set of QS concerns different chemicals than the bioassay battery was based on or can effectively detect, different bioassays may be required to sensitively respond to all regulated chemicals (after a check if none of the implemented bioassays can).

Bioactivity data

What happens if additional bioactivity data become available for chemicals and bioassays?
Testing additional chemicals does not necessarily mean that the bioassay response-BRQ% curves will change, under the assumption that the chemicals included in the current calculations are a representative and data-rich sample of 'all' chemicals that show activity per bioassay. If the newly added AC_{50} values have a distribution similar to the AC_{50}s of already included chemicals, there will be no change in the resulting $BRQ_{\%}$. However, if it concerns a disproportionate number of very potent chemicals, the percentage of potential compliance is structurally a bit higher because such chemicals require low concentrations to elicit a particular bioassay response and have a good chance to have potential maximum concentrations lower than the QS, given the measured bioassay response. The opposite goes for an added collection of AC_{50}s of non-potent chemicals. Before putting the framework into practice, it is advisable to ensure bioactivity data availability for all chemicals under the regulation of interest, as far as possible.

To implement this approach, would bioactivity data be required for all regulated chemicals?
Yes, compliance of chemicals without bioactivity data cannot be evaluated under the framework. Fortunately, AC_{50} (or other) data are generated once and can be repeatedly used in applications of the framework. Unfortunately, some chemicals are not active in any (currently known) bioassay. Such chemicals will by default require chemical analytical quantification. The chemical coverage of the framework is currently limited by the availability of bioactivity data. To enable reliable compliance testing with bioassays, including the identification of specific chemicals that may exceed their QS, all

chemicals with a QS should be tested in commonly used bioassay batteries and results should be uploaded to freely accessible platforms like the NORMAN Network Bioactivity Database (https://www.norman-network.com/nds/badb/index.php).

Some chemicals are regulated by a group approach. Do all individual chemicals in a group require bioactivity data for compliance testing?

If chemicals have a maximum concentration as a group, all individual chemicals will need bioactivity data, with the least potent chemical used as the worst-case scenario in the proposed framework. If the group is individually regulated, all individual chemicals would need bioactivity data to assess compliance. In some cases, regulation has a lenient definition for a group. In the Dutch drinking water regulation, individual pesticides are limited to 0.1 µg/L. Likewise, anthropogenic chemicals have a signaling value of 1µg/L. However, regulation requires only chemical analytical measurement of chemicals that are likely to be present. In that case, the relevant chemicals differ between locations. This means that in the proposed framework, bioactivity data are required only for these 'relevant' chemicals.

Choice of bioassays in a battery

How many bioassays are required for evaluation of all chemicals under regulation?

This will depend on the chemicals in the regulation. An optimal bioassay battery has as little overlap in the chemicals that induce sensitive responses and as broad a chemical space coverage as possible. If regulated chemicals possess unique modes of action, many different bioassays are required to enable sensitive testing of potential concentrations of all chemicals under regulation. Should this be the case, it can be considered to instead perform chemical analyses to rule out non-compliance of such chemicals, rather than applying a prohibitively extensive bioassay battery.

If a single bioassay in practice never indicates full compliance, is it too conservative?

It could be that the bioassay has a low sensitivity to (a subset of) its active chemicals, and high potential concentrations are required to induce a bioassay response. Such a bioassay would not be very useful in compliance testing. However, if chemical analytical tests point out that the sample actually *is* non-compliant, the non-compliance is due to the chemical quality of the tested samples, and the lack of compliance is an indication of poor water quality at the investigated sites.

Will introducing an additional, sensitive bioassay result in a lower BRQ percentage?

Yes. If a bioassay is *not* sensitive to particular chemicals, only high concentrations would trigger the measured bioassay response. This means that the calculated potential maximum concentration is high. If this is higher than the QS, an analytical chemical method would have to confirm if this is indeed non-compliant. A sensitive bioassay would require a low concentration of the particular chemicals, and a measured bioassay response could hence prove that the concentrations are not above the

threshold, indicating that fewer chemicals potentially exceed their threshold, and thus results in a lower $BRQ_{\%}$.

Other questions

How can the $BRQ_{\%}$ framework deal with the potential presence of both agonist and antagonist chemicals for the same receptor-mediated endpoint in a sample?

If the measured bioassay response is a net effect of agonist and antagonist activity, the $BRQ_{\%}$ would be too low. Studies indicate that for surface water and drinking water, such inhibitory effects are not likely. For wastewater or eutrophic water, it has been shown to occur. Studies so far are not clear on the potential net effect in practice, and it remains to be decided how this would fit in the bioassay response-$BRQ_{\%}$ relation. If a significant effect is expected, a safety margin could correct for potential underestimations in wastewater or eutrophic water. Another option is to use effect-directed analyses (EDA), where water extract fractions are tested separately. The antagonist and agonist chemicals would, in that case, be separated before bioassays are employed. Lastly, alternative bioassays that are not receptor-mediated could be included in the bioassay battery.

If the slopes of chemical concentration-response relationships differ, can REP still be used to recalculate bioequivalent concentrations?

For compounds with a similar mode of action, the slopes are generally similar. In the current proof-of-concept, the possibility of different slopes was not evaluated. However, for a final implementation, the difference in slope between chemicals should indeed be considered when calculating bioequivalent concentrations.

Why is thiabendazole the reference compound for all selected bioassays in the paper?

By choosing a single reference compound, the results in Figure 1 in the manuscript can be compared more easily by the reader. The framework works in practice with any suitable reference compound, even if these differ between selected bioassays. For any bioassay, one reference compound can be recalculated to another based on the relative effect potency (REP) of the compound in the bioassay. The choice of reference compound in principle does not impact the calculation of potential concentrations of chemicals.
